# Gastric Microbiota beyond *H. pylori*: An Emerging Critical Character in Gastric Carcinogenesis

**DOI:** 10.3390/biomedicines9111680

**Published:** 2021-11-12

**Authors:** Jun Wen, Harry Cheuk-Hay Lau, Maikel Peppelenbosch, Jun Yu

**Affiliations:** 1 State Key Laboratory of Digestive Disease, Department of Medicine and Therapeutics, Institute of Digestive Disease, Li Ka Shing Institute of Health Sciences, CUHK Shenzhen Research Institute, The Chinese University of Hong Kong, Hong Kong; junwen@link.cuhk.edu.hk (J.W.); harrylau@link.cuhk.edu.hk (H.C.-H.L.); 2 Department of Gastroenterology and Hepatology, Erasmus University Medical Center Rotterdam, Postbus 2040, 3000 CA Rotterdam, The Netherlands; m.peppelenbosch@erasmusmc.nl; 3 Institute of Digestive Disease, Department of Medicine and Therapeutics, Prince of Wales Hospital, The Chinese University of Hong Kong, Shatin, NT, Hong Kong

**Keywords:** gastric microbiota, gastric carcinogenesis, gastric dysbiosis, non-*H. pylori* commensals, microbiota-related carcinogenesis, diagnostic biomarker

## Abstract

Gastric cancer (GC) is one of the global leading causes of cancer death. The association between *Helicobacter pylori*, which is a predominant risk factor for GC, with GC development has been well-studied. Recently, accumulating evidence has demonstrated the presence of a large population of microorganisms other than *H. pylori* in the human stomach. Existing sequencing studies have revealed microbial compositional and functional alterations in patients with GC and highlighted a progressive shift in the gastric microbiota in gastric carcinogenesis with marked enrichments of oral or intestinal commensals. Moreover, using a combination of gastric bacterial signatures, GC patients could be significantly distinguished from patients with gastritis. These findings, therefore, emphasize the importance of a collective microbial community in gastric carcinogenesis. Here, we provide an overview of non-*H. pylori* gastric microbes in gastric carcinogenesis. The molecular mechanisms of gastric microbes-related carcinogenesis and potential clinical applications of gastric microbiota as biomarkers of GC are also explored.

## 1. Introduction

Despite the decreased incidence in recent years, gastric cancer (GC) remains one of the leading causes of cancer-associated death worldwide [1]. GC is a heterogeneous cancer that can be histologically classified into two types, namely an intestinal type (more frequent) and diffuse type (less frequent). Intestinal-type GC has been well-described as a cascade of histological changes in the gastric mucosa from inflammation and atrophy, then intestinal metaplasia (IM), followed by dysplasia, and eventually, to in situ gastric carcinoma and invasive carcinoma [2].

As with many other types of cancer, tumor formation in the stomach is a multifactorial process, and various genetic and environmental factors contribute to the development of GC. Among environmental factors, the role of microorganisms in gastric carcinogenesis has been increasingly recognized over the past decades. Historically, due to the hostile conditions of the gastric ecological niche, the human stomach was considered to be essentially sterile until the discovery of *Helicobacter pylori* in the 1980s [3]. The global attributable fraction of *H. pylori* in non-cardia GC was estimated at 89% in 2008 [4]. In general, *H. pylori* infection has been universally acknowledged as the main risk factor for GC and other gastroduodenal diseases, and its pathogenic mechanisms have been studied in great detail [5,6,7]. In comparison, little is known about the contribution of other microbes that are resident in the stomach to gastric carcinogenesis. Nevertheless, the collective microbial community in the stomach and its role in nontumorous gastric diseases and GC have attracted widespread attention over the past few years [8,9,10,11,12]. In this review, the roles of gastric microbiota in the development of GC and precancerous lesions are summarized with discussions on the mechanisms of microbiota-related carcinogenesis. The potential of utilizing specific microbial features as diagnostic biomarkers of GC is also explored.

### 1.1. Gastric Microbiota in Healthy Individuals

Back in 2000, Monstein et al. were the first to profile the gastric bacterial community other than *H. pylori* by temporal temperature gradient gel electrophoresis of 16S ribosomal DNA (rDNA) amplicons [13]. *Enterococcus*, *Pseudomonas*, *Streptococcus*, *Staphylococcus*, and *Stomatococcus* were identified as the most abundant genera in gastric biopsy specimens from both *H. pylori*-positive patients with gastritis and healthy individuals. Most of these genera are inhabitants of the oral cavity and respiratory tract, thus suggesting the existence of a microbial community indigenous to the gastric microenvironment.

Early evidence from cultivation studies reported that the most abundant bacteria isolated from healthy human stomachs belonged to *Veillonella*, *Lactobacillus*, *Clostridium* [14], *Propionibacterium*, *Streptococcus*, and *Staphylococcus* [15]. In the last decade, the advent of microarray, high-throughput sequencing, and bioinformatic processing have allowed the detection of a wide variety of uncultivated taxa to gain a more comprehensive picture of the gastric microbiota in humans. However, research on the gastric microbiota of healthy populations is still limited, primarily due to the ethical inappropriateness of collecting gastric specimens from healthy individuals. In brief, there is significant inter-subject variability in the gastric microbiota of healthy individuals, with the potential contributing factors including birth mode, geography, diet, lifestyle, antibiotic exposures, use of proton pump inhibitor, and presence of *H. pylori* [16,17,18,19]. In addition, the data available from the literature also varies greatly. Some studies profiled all identified taxa at different taxonomic levels, while other studies only focused on microbes that could discriminate one particular gastric disease from controls, with considerable differences in the definition of “healthy controls”. Nonetheless, representatives of five major phyla were consistently observed in healthy populations, namely *Proteobacteria*, *Firmicutes*, *Bacteroidetes*, *Actinobacteria*, and *Fusobacteria* [18,20,21,22,23], with *Streptococcus*, *Prevotella*, *Veillonella,* and *Rothia* found as the most prevalent genera [24].

### 1.2. Gastric Dysbiosis across Gastric Carcinogenesis

**Figure 1:**
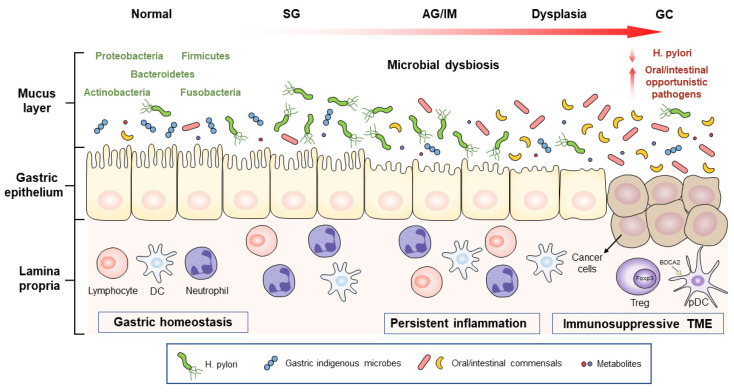
**Schematic representation of the gastric microbial alteration across different stages of gastric carcinogenesis**. *H. pylori* can trigger the initiation of GC, while non-*H. pylori* microorganisms, especially oral or intestinal opportunistic pathogens, are also involved in GC development. These microbes and their metabolites promote gastric carcinogenesis by inducing persistent inflammatory responses and immunosuppressive microenvironments. SG, superficial gastritis; AG, atrophic gastritis; IM, intestinal metaplasia; GC, gastric cancer; DC, dendritic cell; Treg, regulatory T cell; pDC, plasmacytoid dendritic cell; Foxp3, forkhead box P3; BDCA2, blood DC antigen 2; TME, tumor-associated microenvironment.

Significant differences in the composition, function, and interaction network of gastric microbiota exist among the different stages of gastric carcinogenesis, including premalignant lesions (atrophic gastritis (AG), intestinal metaplasia (IM), and dysplasia), and GC [25,26,27,28]. In this section, evidence on the dynamic and gradual alterations in the gastric microbiota, as well as their correlations with GC progression, are discussed and summarized in Figure 1.

#### 1.2.1. Gastric Microbiota in Premalignant Status

Prolonged AG and IM have been well-recognized as premalignant lesions and risk factors of GC [29]. Chronic AG, a condition characterized by the reduced capacity of gastric acid secretion (mostly caused by a persistent infection of *H. pylori*), could promote the survival and colonization of nonindigenous microbes, including opportunistic pathogens from the oral cavity [18,30]. In contrast, Parsons et al. [31] reported that *H. pylori*-induced AG showed a significantly reduced microbial diversity, probably due to the dominance of *H. pylori* lowering the evenness and richness of the gastric microbiota. At the genus level, increased *Streptococcus* and decreased *Prevotella* were observed in patients with *H. pylori*-induced AG [24]. Yet again, inconsistent findings were reported, as another study identified that *Streptococcus* was negatively correlated with *Helicobacter* in patients with *H. pylori*-induced AG [31]. Nevertheless, several bacterial genera, including *Prevotella*, *Tannerella,* and *Treponema*, were consistently found to be depleted in patients with *H. pylori*-induced AG from multiple studies, with *Helicobacter* as the only increased taxon. Moreover, the microbial metabolic pathways could also be altered. For example, an increase in fumarate reductase and reduction in succinate dehydrogenase and glutamate synthase were detected in patients with *H. pylori*-induced AG [31]. Intriguingly, decreased succinate dehydrogenase and succinate accumulation have already been identified in GC, as well as other tumor types, such as colorectal cancer and hepatocellular carcinoma [32].

Autoimmune AG is another etiology of hypochlorhydria, though less prevalent. It is also associated with an increased risk of GC [33,34], particularly linked to the development of type I gastric carcinoid tumors [35]. Patients with autoimmune AG showed similar changes in their metabolic pathways as patients with *H. pylori*-induced AG, including increased abundances of alcohol dehydrogenase, (R-R)-butanediol dehydrogenase, glycerol dehydrogenase, D−arabinitol 4−dehydrogenase, L−iditol 2−dehydrogenase, and myo−inositol 2−dehydrogenase [31]. Whereas the overall composition of the gastric microbiota between patients with autoimmune AG and *H. pylori*-induced AG was found to be distinct. Individuals with autoimmune AG harbored a more diverse bacterial community and significantly higher proportion of *Streptococcus* than patients with *H. pylori*-induced AG and even healthy controls. These findings thus implicate the potential involvement of gastric microbes in gastric carcinogenesis even under the absence of *H. pylori*.

In comparison, much fewer studies have profiled the gastric microbiota in IM, even though IM is well-acknowledged to be the precancerous stage of GC. The gastric microenvironment of patients with IM was once again dominated by *H. pylori* [36]. After the removal of *Helicobacteraceae*-related operative taxonomic units (OTUs), *Actinomyces*, *Prevotella*, and *Streptococcus* were identified in relation to the histopathological diagnosis of multifocal AG with IM [37]. In contrast, the prevalence and abundance of *H. pylori* were significantly lower in GC patients. Moreover, a principal coordinate analysis performed in one study containing subjects with *H. pylori*-associated gastritis, IM, GC, and *H. pylori*-negative controls showed a clear separation in the gastric microbial profiles between GC and other noncancerous groups, as well as a large similarity between *H. pylori*-associated gastritis and IM [38]. A clustering analysis based on UniFrac distance also revealed that the chronic gastritis patients and GC patients were significantly separated, while the IM patients were distributed between the two groups [27]. On the other hand, while there was no significant difference in beta diversity among the patients with superficial gastritis (SG), AG, and IM, the microbial richness estimated by Chao-1 and the alpha diversity was significantly reduced in IM compared to SG or normal controls [26,38]. Additionally, in comparison with SG, seven taxa were significantly enriched in IM, including bacterial species of *Pseudomonas* and *Dyella* [26].

Taken together, these results suggest that *H. pylori* plays a crucial role in the development of chronic inflammation, atrophy, and hypochlorhydria, which creates a favorable growth niche for other opportunistic pathogens to contribute to GC development and progression.

#### 1.2.2. Gastric Microbiota in Gastric Cancer

Similar to the precancerous stages, accumulating evidence has demonstrated the global dysbiosis of gastric microbiota in GC. A gradual alteration in gastric microbial profiles from non-atrophic gastritis (NAG), IM, and intestinal-type GC was reported by Aviles-Jimenez et al., with a steady reduction in the microbial diversity [28]. Likewise, another recent study showed that the gastric microbial richness reduced progressively from healthy controls, NAG, and IM to GC [39]. In contrast, there have been several studies reporting increased microbial richness and diversity in GC tissues as compared to controls [27,40,41,42]. These controversial findings are possibly attributed to the methodological disparity among these studies, as they employed different sequencing platforms, including microarray, pyrosequencing, and next-generation sequencing. Moreover, their study cohorts, sample sizes, and analytic methods also varied widely, which may also account for the discrepant findings.

More recently, an increasing number of studies based on 16S ribosomal RNA (rRNA) gene or shotgun metagenomic sequencing have been performed to depict the gastric microbiota in gastric carcinogenesis [25,26,31,36,37,40,41,43,44,45,46] (Table 1). Despite heterogeneities in ethnic/geographic populations and profiling pipelines, some consistent results among studies have been shown. The enrichment of microbes that originated from the oral cavity or intestines was constantly detected in the gastric microbiota of GC patients [25,26,36,40,41,43,45,47], and these results are consistent with previous datasets using non-sequencing techniques such as terminal restriction fragment length polymorphism analysis and microarray [28,48]. Intriguingly, *H. pylori* has been frequently observed as significantly depleted or even completely absent in the later stages of gastric tumorigenesis, particularly in patients with dysplasia [25,27,39,41,43,46]. Additionally, epidemiological studies have shown the association of *H. pylori* abundance with different GC subtypes. For instance, the incidence of intestinal-type GC exhibits a markedly declining trend, whereas the diffuse type shows an increasing trend in both high- and low-risk areas for GC [49]. The decreased incidence of intestinal-type GC has been principally attributed to improved food preservation and declined *H. pylori* infection rates [1,4]. However, these factors could not explain well the changes in incidence of the diffuse-type GC. Thus, it is of interest to identify whether there is a potential association of non-*H. pylori* microbes with diffuse-type GC, which may possibly explain the changes of its incidence. All these findings imply the involvement of non-*H. pylori* components in the development and progression of GC.

Ferreira et al. investigated the gastric microbiota at the genus level in 135 cases with GC or chronic gastritis by 16S rRNA gene profiling [25]. They identified that GC patients have a dysbiotic microbial community with a significant decrease in microbial diversity compared to patients with chronic gastritis. The enrichment of bacterial genera, which are mainly intestinal commensals, including *Phyllobacterium*, *Achromobacter*, *Citrobacter*, *Lactobacillus*, *Clostridium*, and *Rhodococcus*, together with the depletion of *Helicobacter*, were observed in GC patients. By contrast, *Helicobacter*, *Prevotella,* and *Streptococcus* are the major components in the gastric microbiota of patients with chronic gastritis. Importantly, the results of this study were obtained from a Portuguese discovery cohort with validation by an independent Chinese cohort with 79 GC cases, thus confirming the accuracy of these findings across different geographical populations.

To fully elucidate the alterations in gastric microbiota across GC development, our previous study assessed the microbial composition in gastric mucosal biopsies from 81 cases comprising different histological stages of gastric carcinogenesis, including SG, AG, IM, and GC [26]. Dysbiosis occurred in the gastric microbiota of IM and GC patients with significantly reduced microbial richness in comparison to SG subjects. Lower species richness in GC than in SG was also observed in another metagenomic study [45]. At the genus level, the significant enrichment of 21 bacteria and depletion of 10 bacterial taxa were detected in GC [26]. In agreement with a previous study that showed increased oral-originated bacteria in GC [43], a higher abundance of oral bacteria was observed in GC than in the other three precancerous stages. The enrichment of oral bacterial genera such as *Fusobacterium*, *Haemophilus*, *Veillonella*, *Leptotrichia*, *Dialister*, and *Lactococcus* in GC patients was also highlighted in another study using cDNA from 16S rRNA transcripts [41]. Similarly, Hu et al. [45] reported that the most abundant taxa in GC are commensals or opportunistic pathogens originally colonizing the oral cavity, including *Neisseria*, *Alloprevotella*, and *Aggregatibacter*. Another study published by Chen et al. [40] also illustrated the enrichment of oral bacteria (*Peptostreptococcus*, *Streptococcus*, and *Fusobacterium*) in GC tissues as compared to nontumorous tissues.

In terms of microbial interactions, both cooccurrence and co-exclusive interactions among GC-enriched and GC-depleted bacteria progressively increase from SG, precancerous lesions (AG and IM), and to GC [26]. Microbial interaction networks are also denser, with enhanced complexities in GC patients than in functional dyspepsia controls [41], which could be attributed to the decreased dominance of *H. pylori* and subsequently elevated abundances of non-gastric commensal microbes (e.g., oral taxa) in GC. Collectively, the current findings have provided solid evidence of the predominant presence of oral bacteria in the gastric microbiota of GC patients.

On the other hand, although the widespread application of next-generation sequencing platforms has greatly facilitated microbiome research, it also has its inherent defects. One critical limitation is that almost all recent studies based on 16S rRNA or metagenomic sequencing have reported the composition and relative abundance of gastric microbes but barely considered their absolute abundances or concentrations of relevant microbial products, which are both of great importance in gastric diseases and tumorigenesis. In fact, culture-based research published in the 1970s has already revealed that the number of bacteria detected in a hypochlorhydric stomach was above 1000 times greater than that in normal groups [50]. Hence, it is reasonable to speculate that the increased absolute abundances of some crucial pathogenic microbes are not noticed in modern studies that only analyze the relative abundance. While other less critical bacteria, though with overgrowth, may overshadow the identifications of species that are actually important to GC development.

### 1.3. Interplay of H. pylori with Gastric Microbes in Gastric Carcinogenesis

*H. pylori* infection is known to trigger the initiation and development of GC [6,57,58,59,60]. Persistent *H. pylori* infection leads to a reduced secretion of gastric acid and chronic inflammation and consequently increases gastric pH, which are common features of patients with AG. Given that gastric acid acts as a formidable barrier against the invasion of oral-ingested microorganisms into the gastrointestinal tract, elevated pH may subsequently contribute to the overgrowth of non-*H. pylori* microbes in the gastric ecological niche, whereas reduced gastric acidity weakens its own survival; thus *H. pylori* undergoes a suicidal journey in pathogenesis [61]. Moreover, *H. pylori* can induce the formation of bacterial biofilms to facilitate the colonization of oral bacteria in the stomach [62]. 

With the rapid development of high-throughput next-generation sequencing technology and bioinformatic analysis methodology [63,64], the research on gastric microbiota has been massively accelerated. Most studies have focused on the characterization of the structure and composition of the gastric microbial community regarding the *H. pylori* status, as well as alterations of gastric microbiota in subjects after receiving *H. pylori* eradication [10,31,38,46,65,66,67,68,69,70]. An early study in 2011 performed a high-density 16S rRNA gene microarray on gastric biopsies of subjects with or without *H. pylori* infection [65]. The study reported that the gastric microbiota in *H. pylori*-positive and -negative subjects were both dominated by four phyla (*Proteobacteria*, *Firmicutes*, *Actinobacteria,* and *Bacteroidetes*), whereas their compositions were remarkably different. The presence of *H. pylori* was significantly associated with higher abundances of *Proteobacteria*, *Acidobacteria,* and *Spirochetes* and lower abundances of *Actinobacteria*, *Firmicutes,* and *Bacteroidetes*. Similarly, gastric microbiota in chronic gastritis patients with or without *H. pylori* infection were distinct at the family level, of which relative abundances of *Bradyhizobiaceae*, *Caulobacteraceae*, *Lactobacillaceae*, and *Burkholderiaceae* were significantly increased in *H. pylori*-negative patients [27]. In general, these studies revealed a marked influence of *H. pylori* colonization on the gastric microbial community, hence providing new insights into research on the roles of gastric microbiota other than *H. pylori* in gastric diseases and tumorigenesis.

The abundance of *H. pylori* in later stages of carcinogenesis is consistently reduced in multiple profiling studies, with the least or even undetectable *H. pylori* at the GC stage. Likewise, *H. pylori* seems to be more abundant in the early stages of gastric tumorigenesis (i.e., AG and IM). These findings implicate potential interactions among *H. pylori*, gastric commensal microbes, and translocated oral bacteria. The effects of *H. pylori* on the richness, diversity, and microbial interactions of the gastric microhabitat have therefore been investigated. Li et al. [38] found that *H. pylori*-positive patients showed significant alterations in the structure of gastric mucosal microbiota and reduction in microbial diversity. The negative association of the abundance of *H. pylori* with microbial diversity and interaction networks was also reported in another two studies [31,68]. Similarly, our previous publication revealed that interactions among gastric microbes were drastically weaker in *H. pylori*-positive than *H. pylori*-negative specimens across all stages of gastric tumorigenesis (SG, AG, and IM) [26]. In particular, distinct representative taxa could interact with *H. pylori* at different stages of gastric tumorigenesis, further indicating the critical role of *H. pylori* in altering microbial interactions. Notably, Liu et al. [46] performed 16S rRNA gene sequencing on normal, peri-tumoral, and intra-tumoral stomach tissues from 276 GC patients and identified that higher *H. pylori* colonization significantly impacted the whole structure of gastric microbiota in both normal and peri-tumoral microenvironments [40,46].

The association of *H. pylori* and other microbes in GC development was also elucidated by restored gastric microbiota after *H. pylori* eradication. Whilst well-established evidence supports the preventive effect of *H. pylori* eradication against GC prevention, Li et al. [38] indicated that *H. pylori* eradication could play a role in preventing GC, possibly by restoring the dysbiotic gastric microbiota. In our previous study, 404 gastric biopsy samples from *H. pylori*-positive patients before and after one-year *H. pylori* eradication were analyzed [71]. After one year of *H. pylori* eradication, the patients had a significant increase in bacterial diversity with distinct composition of gastric microbiota compared to those without eradication treatment, which could be attributed to the absence of *H. pylori*, the dominant taxa before treatment. A significant decrease in cooccurrence interactions in the microbial ecological network was also observed after *H. pylori* eradication, whereas *H. pylori* had predominant co-exclusive correlations with other gastric microbes in the placebo group, indicating the inverse interplay between *H. pylori* and other gastric microbes. In the meanwhile, the depletion of probiotic *Faecalibacterium* was observed in subjects developing AG after *H. pylori* eradication. Further metagenomic functional predictions suggested an increase in energy generation and stress adaptation in the inflammation-related microbiota, as well as an enrichment of KEGG ontologies encoding bacterial virulent factors. This study elucidated the contribution of non-*H. pylori* microbes in the persistence and progression of the precancerous status after *H. pylori* eradication and rendered new clues for utilizing the microbiota as potential interventional targets for the prevention and therapeutic modulation of gastric carcinogenesis.

### 1.4. Mechanisms of Gastric Microbes in Gastric Carcinogenesis

Solid evidence has revealed the link between gastric commensal microbes other than *H. pylori* and the development of GC. However, much fewer studies have deciphered the mechanisms of non-*H. pylori* in promoting carcinogenesis. In general, from the limited amount of animal studies, gastric microbes could induce DNA damage, inflammation, and immunosuppression to promote gastric carcinogenesis.

#### 1.4.1. The Involvement of Non-*H. pylori* Commensals in GC Development

Hypergastrinemic insulin-gastrin (INS-GAS) mice are one of the most commonly used mouse models of gastric carcinoma that can develop spontaneous AG and gastrointestinal intraepithelial neoplasia (GIN) 6 months after *H. pylori* infection. Using this mouse model, an early study showed that *H. pylori*-monocolonized mice exhibited a delayed development of GIN compared to *H. pylori*-infected specific pathogen-free mice [72]. These findings suggested the involvement of a complex gastric microbial community other than *H. pylori* in promoting gastric carcinogenesis. Another study by Lertpiriyapong et al. [73] found that colonizing INS-GAS mice with a combination of microbes limited to only three species of commensal bacteria, including *Clostridium* species, *Bacteroides* species, and *Lactobacillus murinus*, had equivalent gastritis, atrophy, and dysplasia to those colonized with complex intestinal microbiota, and both groups were independent from *H. pylori* infection. Further *H. pylori* colonization promoted invasive GIN to a similar extent in mice co-colonized with restricted commensals or diverse microbiota. In these *H. pylori*-infected mice, the enrichment of *Lactobacillus* species was both observed in mice with restricted colonization of commensals and colonization of complex bacteria, which were both linked to the depleted abundance of *H. pylori*. Consistent with previous studies, an inverse association of the abundance of *H. pylori* and microbial diversity was also observed in this study. These results altogether indicated that, in the setting of *H. pylori*-induced gastritis and atrophy, restricted commensal bacteria are sufficient for progression to GIN comparable with complex microbiota.

#### 1.4.2. Non-*H. pylori* Commensals Produce Carcinogens to Contribute GC Development

Another mechanism of non-*H. pylori* gastric microbes in gastric carcinogenesis is that nitrate-reducing bacteria could contribute to gastric malignancy by increasing the concentrations of nitrite and N-nitroso compounds in the stomach. Ferreira et al. reported that, when compared to chronic gastritis, the functional composition of gastric microbiota in GC displayed enhanced nitrate reductase and nitrite reductase functions, which could promote the generation of nitrite (precursor of carcinogenic N-nitroso compounds) and nitric oxide (a DNA damage inducer), respectively [25]. These data suggest the contribution of a gastric microbial community to genotoxicity in GC. Consistently, another study revealed that proportions of nitrosating and nitrate-reducing non-*H. pylori* gastric bacteria were two times higher in the GC group than in the controls [51]. Besides, *Escherichia–Shigella*, *Lactobacillus*, and *Nitrospirae*, which are all known to contribute to the metabolism of nitrate or nitrite, were also reported to be enriched in GC [67]. Therefore, these bacteria could play a role in gastric carcinogenesis by promoting the production of carcinogenic N-nitroso compounds. Additionally, several more pathways, including carbohydrate metabolism, amino acid metabolism, nucleotide metabolism, energy metabolism, and DNA replication and repair, were reported to be significantly enriched in GC [26,41,43].

#### 1.4.3. Non-*H. pylori* Commensals Induce Inflammatory Response and Immunosuppressive Microenvironment to Contribute GC Development

There are a few concordant findings on the role of specific species in gastric carcinogenesis. *Propionibacterium acnes* was significantly enriched in the GC microenvironment in two independent studies and identified as a strong risk factor for GC development [46,52]. *P. acnes* was previously detected as a trigger for corpus-dominant lymphocytic gastritis by activating the natural killer group 2 member D (NKG2D) system and upregulating the secretion of proinflammatory cytokine interleukin (IL)-15 [74]. Inducing the expression of NKG2D ligands on stressed cells was shown to be a consequence of the DNA damage response. The current evidence also illustrates that microbial dysbiosis can trigger a DNA damage response in host cells such as epithelial cells, which may result in prolonged inflammatory responses, as well as tumorigenesis [75]. On the other hand, some controversial findings remain to be addressed. For instance, patients with high levels of *Prevotella copri* showed a significantly increased risk for GC in a case–control study consisting of 268 GC patients and 288 healthy controls [52], while its abundance was significantly reduced in tumor tissues in another cohort of 276 GC patients [46]. *P. copri* could promote chronic inflammatory conditions via enhancing the resistance to host-derived reactive oxygen species and generating redox proteins, whereas it is also able to ameliorate glucose metabolism in a beneficial manner [76,77]. Such heterogeneous results are possibly due to the high strain variabilities across individuals. Thus, further mechanistic studies are required to verify the role of *P. copri* in gastric carcinogenesis.

The infamous *Fusobacterium nucleatum* has been well-studied in the carcinogenesis of colorectal cancer, while a recent study also showed its diagnostic potential in GC [36]. The interaction between *F. nucleatum* adhesins (e.g., FadA and Fap2) and host cells can induce inflammatory responses and immune suppression [78,79]. The adhesion from FadA of *F. nucleatum* to E-cadherin of intestinal epithelial cells drives the activation of the Wnt/β-catenin pathway to promote the proliferation of tumor cells [80]. *F. nucleatum* can also activate the NF-κB pathway to stimulate the production and release of inflammatory cytokines such as IL-1β, IL-6, IL-8, and TNF, thereby creating a proinflammatory microenvironment that favors tumor development [81]. Additionally, *F. nucleatum* can modulate the tumor microenvironment by suppressing antitumor immune responses. The binding of *F. nucleatum* adhesin Fap2 to the TIGIT receptor expressed on natural killer cells and other tumor-infiltrating T cells inhibits the cytotoxicity of these immune cells [82]. Although direct mechanistic evidence of how *F. nucleatum* contributes to gastric carcinogenesis is lacking, *F. nucleatum* may possibly promote the development of GC through inducing inflammation and suppressing the host antitumor immunity.

Additionally, Rolig et al. [83] treated C57BL/6 mice with antibiotics prior to *H. pylori* infection, and significantly reduced *H. pylori*-induced inflammation occurred in these microbiota-depleted mice. Notably, the infiltration of CD4^+^ T cells and levels of IFN-γ transcript in the stomach were decreased in *H. pylori*-infected mice with an antibiotic pretreatment. Given the declined abundance of *H. pylori* during progression from gastritis and IM to GC [36], it is reasonable to hypothesize that the enrichment of some bacteria such as *Lactobacillus* species may play an essential role in outcompeting and inhibiting *H. pylori* colonization, thereby promoting the persistent inflammation and progression of GC. Furthermore, our recent study revealed that the enrichment of *Acinetobacter, Ralstonia, Actinobacillus*, and *Erwinia* was correlated with persistent inflammation after one year of *H. pylori* eradication, and oral microbes, including *Granulicatella, Peptostreptococcus, Streptococcus, Rothia, Parvimonas*, and *Prevotella*, were also positively correlated with persistent or emerged atrophy or IM after treatment [71].

In 2020, a considerable impact of the gut microbiota on the human immune system was reported [84]. Likewise, a preliminary study explored the association between gastric mucosal microbiota and immunosuppressive cells, the critical components of the tumor microenvironment [53]. The enrichment of regulatory T cells (Tregs) and BDCA2^+^ plasmacytoid dendritic cells (pDCs), which can both contribute to the immunosuppressive microenvironment in GC [85,86], were observed in intra-tumoral and peri-tumoral tissues. Meanwhile, the composition, diversity, and function of the gastric microbiota were also altered significantly in intra-tumoral tissues. A correlation analysis revealed that an abundance of *Stenotrophomonas* and *Selenomonas* is positively correlated with pDCs and Tregs, respectively, whereas abundances of *Comamonas* and *Gaiella* are negatively correlated with pDCs and Tregs, respectively. These findings indicate that alterations in the gastric microbiota could participate in the modulation of immune cell populations, contributing to an immunosuppressive microenvironment of GC. Nevertheless, further research is still needed to elucidate the detailed mechanism by which these certain microbes modulate the tumor-immune microenvironment.

### 1.5. Clinical Application of Gastric Microbes for Diagnosis of Gastric Cancer

Given the identification of specific gastric microbes that are enriched/depleted in patients with GC, these microbes could be employed as potential biomarkers for GC screening. A gastric microbiota-based diagnostic measurement known as the microbial dysbiosis index (MDI) was developed, which utilizes and combines GC-enriched and -depleted taxa for discriminating GC patients from patients with chronic gastritis [25]. MDI exhibited excellent sensitivity and specificity in identifying GC, achieving areas under the receiver operating characteristic curves (AUROC) of 0.89–0.91. 

Our previous study identified five GC-enriched bacteria with significant centralities among the microbial interaction network of GC [26]. This core set of bacteria includes *Peptostreptococcus stomatis*, *Streptococcus anginosus*, *Parvimonas micra*, *Slackia exigua,* and *Dialister pneumosintes*, all of which are commensals in the human oral microbiome. This set of bacterial markers was employed to distinguish GC patients from patients with SG, showing a confident AUROC of 0.82, while, in another study comprising cases of gastritis, IM, and GC, a combination of five GC-enriched bacteria, including *Clostridium colicanis*, *Fusobacterium canifelinum*, *F. nucleatum*, *Lactobacillus gasseri*, and *Lactobacillus reuteri*, also showed sufficient power to discriminate GC patients from patients with noncancerous gastric diseases with excellent specificity (100%) and good sensitivity (70%) [36]. The diagnostic performance of using only two of the above five bacteria (*C. colicanis* and *F. nucleatum*) exhibited 100% sensitivity for the detection of GC. Strikingly, both studies had large sample sizes, hence providing firm evidence for utilizing these bacterial markers to accurately identify GC patients. Overall, with the advances in microbial profiling technology, recent publications have emphasized the importance of a collective microbial community rather than single bacterial species in gastric carcinogenesis, as well as its clinical potential in diagnosing GC.

Although the above-mentioned microbes may serve as potential diagnostic markers for GC, it does not seem feasible to replace traditional screening or diagnostic methods with a gastric microbial examination. The main reason is the difficulty in collecting gastric microbiota, as gastric biopsy specimens are needed to obtain a thorough invasive gastroscopic examination. In comparison, a microbiota-based diagnosis is much more suitable for screening colorectal cancer, since this can be done without any invasive approach simply by examining the fecal samples of patients. On the other hand, if a gastroscopy with the collection of gastric mucosal biopsies has already been performed, it appears to be unreasonable and unnecessary to use any additional diagnostic approaches apart from histopathological tests. Hence, the therapeutic potential of targeting gastric microbiota to prevent or alleviate GC development could possibly be an alternative clinical application. Nevertheless, it is essential to elucidate the role and detailed mechanism of GC-associated bacterial species identified in previous sequencing studies. If any microbe is found to be an oncogenic pathogen and plays a key role in the development of GC, the elimination of this microbe, just like *H. pylori* eradication, may protect patients, especially those with gastric premalignant lesions, from progressing to GC.

### 1.6. Further Perspectives

There are now increasing studies with high-throughput sequencing to depict the roles of gastric microbiota in GC. This research direction has become more extensive, with increasingly detailed discussions. However, due to the wide variations among individuals with diverse genetic or environmental backgrounds, approaches for sampling, sample storage, DNA extraction methods, sequencing platform and depth, primer design, and data analytic pipelines, the results of many microbiota sequencing studies lack comparability [87,88]. Moreover, these studies mostly focus on profiling the gastric microbiota, while the mechanism of how the altered microbiota contributes to carcinogenesis is much less acknowledged. Additionally, given that most publications on gastric microbiota are based on 16S rRNA gene sequencing, relevant research on other components in the gastric microbiota, including fungi and viruses, is scarce. For instance, we previously reported the importance of dysbiosis among gastric fungi in gastric carcinogenesis [26]. Taken together, as compared to the intestinal microbiota, many areas of gastric microbe-associated carcinogenesis require extensive investigation, and the potential clinical applications of targeting gastric microbiota for the diagnosis, prevention, and treatment of GC still require further investigation.

## Figures and Tables

**Table 1:** Characteristics of sequencing-based studies on human gastric microbiota in gastric carcinogenesis.

First Author	Country	Sample Size	Sample Type	Sequencing Method	Key Results
Dicksve [48] (2009)	Sweden	Dyspeptic controls, *n* = 5; GC, *n* = 10	Gastric biopsies	16S rRNA gene seq	*Streptococcus*, *Lactobacillus*, *Veillonella,* and *Prevotella* dominated GC microbiota, with relatively low abundance of *H. pylori*.
Aviles-Jimenez [28] (2014)	Mexico	NAG, *n* = 5; IM, *n* = 5; GC, *n* = 5	Gastric biopsies	16S rRNA gene microarray	Steady reduction in gastric bacterial diversity observed from NAG, IM, and to intestinal-type GC.
Eun [27] (2014)	South Korea	Chronic gastritis, *n* = 10; IM, *n* = 10; GC, *n* = 11	Gastric biopsies	16S rRNA gene seq	Depleted *Helicobacteraceae* and enriched *Streptococcaceae* in GC compared with chronic gastritis and IM.
Jo [51] (2016)	South Korea	*H. pylori*-negative controls, *n* = 13; *H. pylori*-negative GC, *n* = 19; *H. pylori*-positive controls, *n* = 16; *H. pylori*-positive GC, *n* = 15	Gastric biopsies	16S rRNA gene seq	Enriched nitrosating or nitrate-reducing bacteria other than *H. pylori* in GC.
Castaño-Rodríguez [41] (2017)	Singapore, Malaysia	Functional dyspepsia, *n* = 20; GC, *n* = 12	Gastric biopsies	16S rRNA gene seq	Increased richness and phylogenetic diversity in GC; *Lactococcus*, *Veilonella*, *Fusobacterium,* and *Leptotrichia* enriched in GC.
Yu [43] (2017)	China, Mexico	GC, *n* = 80 (China); GC, *n* = 80 (Mexico)	Surgical resection specimens	16S rRNA gene seq	*H. pylori* as the most abundant microbes in nontumor tissue in GC, followed by oral-associated bacteria; Gastric microbiota composition did not differ by *H. pylori* status or gastric anatomic sites but did differ between paired tumor and nontumor tissues.
Coker [26] (2018)	China	Discovery cohort: SG, *n* = 21; AG, *n* = 23; IM, *n* = 17; GC, *n* = 20; Validation cohort: SG, *n* = 56; AG, *n* = 51; GC, *n* = 19	Gastric biopsies	16S rRNA gene seq	Significant dysbiosis in gastric microbiota of IM and GC compared with SG; Oral microbes including *P. stomatis, S. anginosus, P. micra, S. exigua,* and *D. pneumosintes* were top enriched in GC and could be used as biomarkers to distinguish GC from SG.
Ferreira [25] (2018)	Portugal	Discovery cohort: Chronic gastritis, *n* = 81; GC, *n* = 54; Validation cohort: Chronic gastritis, *n* = 15 GC, *n* = 23 (Portugal); GC, *n* = 79 (China); GC, *n* = 53 (Mexico)	Gastric biopsies	16S rRNA gene seq	Decreased microbial diversity; depleted *Helicobacter;* and enriched intestinal commensals, including *Phyllobacterium*, *Achromobacter*, *Citrobacter*, *Clostridium, Lactobacillus,* and *Rhodococcus* in GC; Combining the above taxa could excellently discriminate patients with GC from those with chronic gastritis.
Hu [45] (2018)	China	SG, *n* = 5; GC, *n* = 6	Gastric wash samples	Shotgun metagenomic seq	Decreased microbial richness in GC; Oral commensals or opportunistic pathogens including *Neisseria*, *Alloprevotella,* and *Aggregatibacter* were the most representative taxa in GC.
Hsieh [36] (2018)	Taiwan	Gastritis, *n* = 9; IM, *n* = 7; GC, *n* = 11	Gastric biopsies	16S rRNA gene seq	Significantly depleted *H. pylori* in GC; *Clostridium*, *Fusobacterium*, and *Lactobacillus* enriched in GC.
Chen [40] (2019)	China	62 pairs of GC tumor and adjacent nontumor tissues	Gastric biopsies	16S rRNA gene seq	Increased microbial richness and diversity in tumor tissues; Oral bacteria, including *Peptostreptococcus*, *Streptococcus*, and *Fusobacterium* enriched in tumor tissues; actic acid-producing bacteria (e.g., *L. lactis* and *L. brevis*) in nontumor tissues.
Gunathilake [52] (2019)	South Korea	Healthy controls, *n* = 288; Early GC, *n* = 268	Gastric biopsies	16S rRNA gene seq	Significantly higher risk for GC in patients with high levels of *H. pylori*, *Propionibacterium acnes,* and *Prevotella copri*; *L. lactis* as a protective factor for GC development.
Ling [53] (2019)	China	GC, *n* = 64	Surgical resection specimens	16S rRNA gene seq	Abundances of *Stenotrophomonas* and *Selenomonas* positively correlated with pDCs and Tregs, respectively; Abundances of *Comamonas* and *Gaiella* negatively correlated with pDCs and Tregs, respectively.
Liu [46] (2019)	China	GC, *n* = 276	Surgical resection specimens	16S rRNA gene seq	Bacterial richness reduced in peri-tumoral and tumoral microhabitats; Depleted *H. pylori*, *P. copri,* and *B. uniformis* but enriched *P. melaninogenica*, *S. anginosus,* and *P. acnes* in tumoral microhabitats.
Park [54] (2019)	Korea	SG, *n* = 62 IM, *n* = 21 GC, *n* = 55	Gastric biopsies	16S rRNA gene seq	Enriched *Rhizobiales* in patients with *H. pylori*-negative IM compared to *H. pylori*-negative SG or GC; Abundances of *Rhizobiales* and *Neisseriaceae* gradually increased as *H. pylori* decreased.
Wang [55] (2020)	China	Chronic gastritis, *n* = 60 Early GC, *n* = 30 Advanced GC, *n* = 30	Gastric biopsies	16S rRNA gene seq	Distinct microbial community structure and composition between early and advanced GC
Wang [39] (2020)	China	Healthy controls, *n* = 30; NAG, *n* = 21; IM, *n* = 27; GIN, *n* = 25; GC, *n* = 29	Gastric biopsies	16S rRNA gene seq	Progressive decrease in gastric microbial richness and alpha diversity from healthy controls, NAG, IM, intraepithelial neoplasia, and to GC; *Nitrospirae*, a nitrite-oxidizing phylum, depleted during neoplastic progression.
Ravegnini [56] (2020)	Italy	Signet-ring cell carcinoma, *n* = 10 Adenocarcinoma, *n* = 10	Surgical specimens	16S rRNA gene seq	Enriched *Prevotella*, *Fusobacterium*, *Actinomyces*, *Stenotrophomonas,* and *Roseburia* in signet-ring cell carcinoma; Increased *Halomonas*, *Shewanella*, *Pantoea*, *Faecalibacterium,* and *Neoasaia* in adenocarcinoma.
Gantuya [42] (2020)	Mongolia	Healthy controls, *n* = 20; Gastritis, *n* = 20; AG, *n* = 40; IM, *n* = 40; GC, *n* = 48	Gastric biopsies	16S rRNA gene seq	Highest bacterial alpha diversity in healthy controls, followed by IM and GC with the least in gastritis and AG; *Carnobacterium*, *Glutamicibacter*, *Paeniglutamicibacter*, *Fusobacterium,* and *Parvimonas* associated with GC, regardless of *H. pylori* infection status.
Gunathilake [23] (2021)	Korea	Healthy controls, *n* = 288; GC, *n* = 268	Gastric biopsies	16S rRNA gene seq	Bacterial richness and evenness negatively correlated with *Helicobacter* species abundance, while MDI positively correlated with *Helicobacter* species abundance in both GC and controls; Higher abundance of *Actinobacteria* species associated with increased risk of GC.

Note: GC, gastric cancer; NAG, non-atrophic gastritis; AG, atrophic gastritis; IM, intestinal metaplasia; SG, superficial gastritis; pDCs, plasmacytoid dendritic cells; Tregs, regulatory T cells; MDI, microbial dysbiosis index; rRNA, ribosomal RNA.

## Data Availability

The data is available within the article.
